# Spatial Memory Training Counteracts Hippocampal GIRK Channel Decrease in the Transgenic APP_Sw,Ind_ J9 Alzheimer’s Disease Mouse Model

**DOI:** 10.3390/ijms232113444

**Published:** 2022-11-03

**Authors:** Sara Temprano-Carazo, Ana Contreras, Carlos A. Saura, Juan D. Navarro-López, Lydia Jiménez-Díaz

**Affiliations:** 1 Neurophysiology & Behavior Lab, Centro Regional de Investigaciones Biomédicas (CRIB), School of Medicine of Ciudad Real, University of Castilla-La Mancha, 13079 Ciudad Real, Spain; 2 Institut de Neurociències, Department de Bioquímica i Biología Molecular, Universitat Autònoma de Barcelona, 08193 Barcelona, Spain; 3 Centro de Investigación Biomédica en Red Enfermedades Neurodegenerativas (CIBERNED), 28029 Madrid, Spain

**Keywords:** hippocampus, GIRK, Alzheimer’s disease, APP_Sw,Ind_ J9, spatial memory, excitatory/inhibitory imbalance

## Abstract

G-protein-gated inwardly rectifying potassium (GIRK) channels are critical determinants of neuronal excitability. They have been proposed as potential targets to restore excitatory/inhibitory balance in acute amyloidosis models, where hyperexcitability is a hallmark. However, the role of GIRK signaling in transgenic mice models of Alzheimer’s disease (AD) is largely unknown. Here, we study whether progressive amyloid-β (Aβ) accumulation in the hippocampus during aging alters GIRK channel expression in mutant β-amyloid precursor protein (APP_Sw,Ind_ J9) transgenic AD mice. Additionally, we examine the impact of spatial memory training in a hippocampal-dependent task, on protein expression of GIRK subunits and Regulator of G-protein signaling 7 (RGS7) in the hippocampus of APP_Sw,Ind_ J9 mice. Firstly, we found a reduction in GIRK2 expression (the main neuronal GIRK channels subunit) in the hippocampus of 6-month-old APP_Sw,Ind_ J9 mice. Moreover, we found an aging effect on GIRK2 and GIRK3 subunits in both wild type (WT) and APP_Sw,Ind_ J9 mice. Finally, when 6-month-old animals were challenged to a spatial memory training, GIRK2 expression in the APP_Sw,Ind_ J9 mice were normalized to WT levels. Together, our results support the evidence that GIRK2 could account for the excitatory/inhibitory neurotransmission imbalance found in AD models, and training in a cognitive hippocampal dependent task may have therapeutic benefits of reversing this effect and lessen early AD deficits.

## 1. Introduction

Over 55 million people worldwide have been diagnosed with dementia, of which 60–70% suffer from Alzheimer’s disease (AD) [1]. AD is characterized by extracellular accumulation of β-amyloid (Aβ) plaques and intracellular neurofibrillary tangles of hyperphosphorylated tau, leading to a progressive neurodegeneration and memory loss. Despite the extended research in the topic, no current therapeutic disease-modifying intervention exists (for a review, see [2]).

Amyloid precursor protein (APP) transgenic mice show pathological changes resembling those of AD patients [3,4,5]. Biochemical and histological studies have shown that 2-month-old APP_Sw,Ind_ J9 transgenic mice do not exhibit Aβ in the hippocampus, which begins to be detectable at 6 months of age, and by 12–18 months it is accumulated in senile plaques. Moreover, these transgenic mice show spatial memory deficits at 6 months in hippocampal-dependent memory tasks, such as Morris water maze (MWM) [6,7]. Therefore, the progression of biochemical, histological and behavioral changes over time make APP_Sw,Ind_ J9 mice an excellent murine model to compare early and late disease stages.

Hippocampal dysfunction is one of the first alterations in early stages of AD, such as excitatory/inhibitory imbalance, synaptic plasticity disruption and memory deficits [2,8]. Given the proven neuronal hyperactivity in AD animal models [9], G-protein-gated inwardly rectifying potassium (GIRK) channels have emerged as potential targets to regulate neuronal activity, based on their inhibitory role [10,11,12]. GIRK channels are a family of K^+^ channels activated by a variety of G-protein-coupled receptors, such as GABA, dopamine, serotonin or adenosine [12,13,14]. They form tetrameric units, assembled by a combination of their subunits GIRK1–GIRK4. In the brain, GIRK1, GIRK2 and GIRK3 are widely expressed throughout different areas, while GIRK4 subunit is classically related to the heart and its neuronal expression is limited [15]. Moreover, GIRK2 seems to be necessary for the correct function of these channels, and GIRK1/GIRK2 heteromers have been identified as the main GIRK channels in neurons [12,16].

Additionally, GIRK function and location depends on the effect of different modulators. Among those, the Regulator of G-protein signaling 7 (RSG7) is of special interest. It functions as a GTPase activating protein (GAP) that accelerates G-protein inactivation. Accordingly, mice lacking RGS7 show profoundly slow GIRK channel deactivation kinetics [17], disruption of inhibitory forms of synaptic plasticity and deficits in learning and memory [18].

Previous data from our group showed that in vitro acute incubation with Aβ soluble species induces depolarization of CA3 hippocampal pyramidal neurons by a loss-of-function of GIRK channels [19] and dysregulation of GIRK subunits genes (specifically, genes encoding GIRK2, GIRK3 and GIRK4) [20]. Furthermore, increasing GIRK activity restores hippocampal Long-Term Potentiation (LTP) and memory deficits induces by Aβ in an in vivo mouse model of amyloidosis [21,22,23]. However, all this promising data have been obtained in an intracerebroventricular murine model of early amyloidosis, when Aβ is soluble rather than accumulated. A recent study has provided evidence of GIRK redistribution from the plasma membrane to intracellular sites and pre- and post-synaptic reduction in GIRK2 channels in two transgenic AD models, P301S and APP/PS1 [24]. Nevertheless, it is unknown whether GIRK subunits levels are early altered in the brain of mice developing AD pathological changes that include progressive age dependent Aβ accumulation. Furthermore, it is well-established that periodic cognitive training on a hippocampal-dependent memory task may mitigate memory deficits observed in AD, both in rodents [7,25,26] and humans [27,28], as well as attenuate Aβ deposition and enhance adult hippocampal neurogenesis [29]. Accordingly, training in a spatial memory task might be able to reverse early alterations in GIRK expression caused by Aβ-overexpression, with the subsequent recovery of hippocampal function.

Thus, the aim of the present study was to determine the effect of Aβ accumulation (due to genotype and age) and training on the protein expression of GIRK subunits and its modulator, in the hippocampus of APP_Sw,Ind_ J9 transgenic mice.

## 2. Results

**Figure 1:**
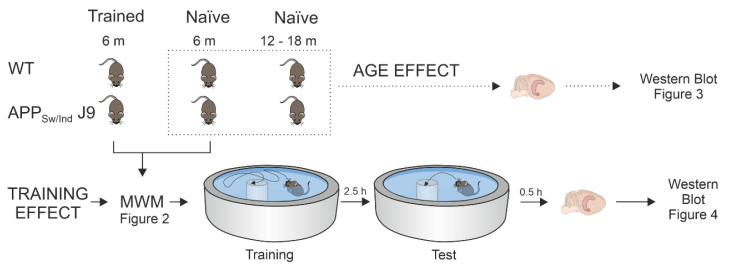
Experimental design showing number of animals for each condition, representation of training in the Morris Water Maze (MWM), hippocampal dissection for Western blot analysis, and corresponding figure numbers.

**Figure 2:**
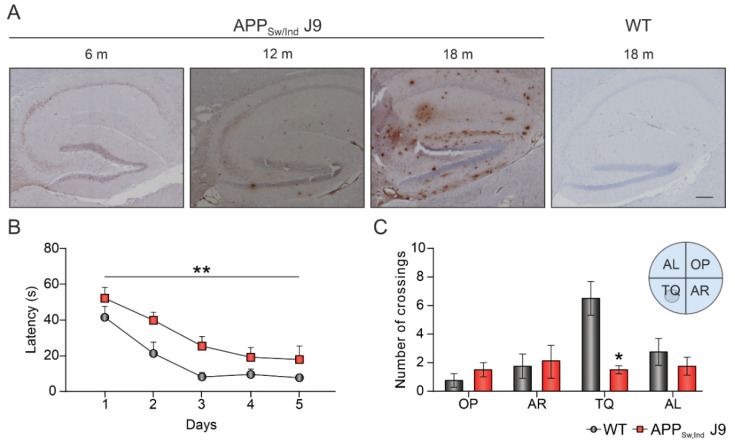
Age-dependent progression of amyloid pathology. (**A**) Age-dependent amyloid accumulation in the hippocampus of APP_Sw,Ind_ J9 mice at 6, 12 and 18 months of age. Brain sections were stained with an anti-Aβ 6E10 antibody. Scale bar: 250 µm. m, months; h, hours; WT, wild type. (**B**) Latency to find the hidden platform in the MWM during 5 days of training. Data is expressed as mean ± SEM. s, seconds. ** *p* < 0.01. (**C**) Number of crossings to the different quadrants during the memory test. Data is expressed as mean ± SEM. TQ, target quadrant; OP, opposite; AR, adjacent right; AL, adjacent left. * *p* < 0.05.

The experimental design of this study is detailed in Figure 1. Firstly, we examined the protein levels of GIRK channel subunits and RGS7 in the hippocampus of adult APP_Sw,Ind_ J9 transgenic mice at early (6 months) and late (12–18 months) pathological stages that differ in hippocampal Aβ accumulation levels [7]. As shown in Figure 2A, APP_Sw,Ind_ J9 mice showed intracellular Aβ accumulation in the hippocampus at 6 months and amyloid plaques at 12–18 months. Thus, 6 months of age in this murine model is an adequate choice to study early disease stages vs. late ones (12–18 months). In addition, APP_Sw,Ind_ J9 mice showed a memory impairment at 6 months of age, as data from the MWM showed a higher latency to find the platform (Figure 2B; F_(1,6)_ = 21.23; *p* = 0.0037) in the transgenic group compared to the control, as well as a lower number of crossings specifically to the target quadrant (TQ; Figure 2C; t_(3)_ = 5.00; *p* = 0.0154).

**Figure 3:**
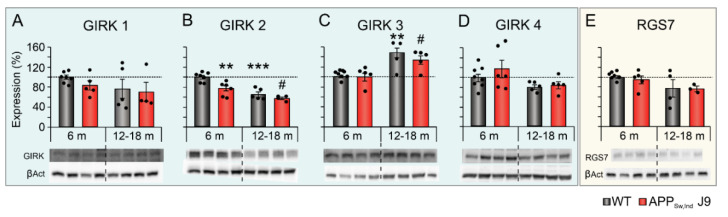
Effect of genotype and age on hippocampal levels of GIRK channel subunits and modulator. Protein levels of GIRK channel subunits (1–4) and modulator (RGS7) in the hippocampus of non-transgenic (WT) and APP_Sw,Ind_ J9 mice at 6 and 12–18 months of age. (**A**) GIRK1, (**B**) GIRK2, (**C**) GIRK3, (**D**) GIRK4 and (**E**) RGS7. Data is expressed as percentage relative to 6-month-old WT mice (100%). ** *p* < 0.01, *** *p* < 0.001 vs. WT 6-month-old; # *p* < 0.05 vs. APP_Sw,Ind_ J9 6-month-old. m, months; βAct, β actin.

An age effect was observed in the expression of GIRK2 (F_(1,18)_ = 40.260; *p* < 0.001) and GIRK3 (F_(1,18)_ = 19.775; *p* < 0.001) in both WT and APP_Sw,Ind_ J9 mice. These effects were opposite, as GIRK2 expression was decreased (Figure 3B; WT: t_(10)_ = 5.824; *p* < 0.001; APP_Sw,Ind_ J9: t_(8)_ = 3.316; *p* = 0.0106) and GIRK3 was increased (Figure 3C; WT: t_(10)_ = 3.412; *p* = 0.0066; APP_Sw,Ind_ J9: t_(8)_ = 2.889; *p* = 0.0202) in aged WT and APP_Sw,Ind_ J9 mice. No statistical differences were found in hippocampal levels of GIRK1, GIRK4 and RGS7 in the experimental groups during aging (Figure 3A,D,E). These data suggest that age, regardless of the genotype, varies the expression of specific GIRK subunits.

However, quantitative analyses of hippocampal lysates showed a significant effect of genotype in the expression of GIRK2 (F_(1,18)_ = 11.645; *p* = 0.003). As shown in Figure 3B, GIRK2 was downregulated in 6-month-old APP_Sw,Ind_ J9 mice compared to age-matched controls (t_(11)_ = 3.824; *p* = 0.0028). This data indicates that cerebral Aβ accumulation modulates GIRK channel expression and accelerate its decrease compared to WT animals.

**Figure 4:**
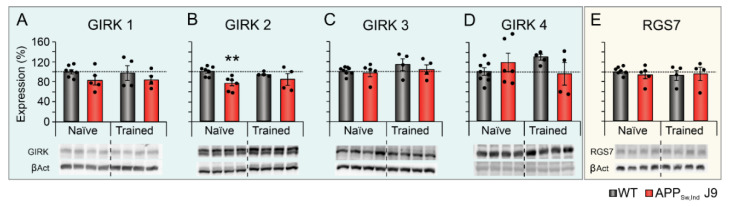
Effect of genotype and spatial memory training on hippocampal levels of GIRK channel subunits and modulator. Protein levels of GIRK channel subunits (1–4) and modulator (RGS7) in the hippocampus of naïve and memory-trained non transgenic (WT) and APP_Sw,Ind_ J9 mice at 6 months of age. (**A**) GIRK1, (**B**) GIRK2, (**C**) GIRK3, (**D**) GIRK4 and (**E**) RGS7. Data is expressed as percentage relative to untrained naïve WT mice (100%). ** *p* < 0.01 vs. naïve WT. βAct, β actin.

Next, we analyzed the effect of spatial memory training in the MWM on the levels of GIRK subunits and RGS7 (Figure 1). As shown in Figure 2B,C, at 6 months of age, APP_Sw,Ind_ J9 mice showed higher latencies to find the hidden platform and reduced number of target platform crossings and target platform occupancy in the probe trial [7]. No significant changes were found in GIRK subunits and their modulator in naïve vs. trained animals (Figure 4A–E), but our results showed once again a genotype effect (decrease) in the expression of GIRK2 (Figure 4B; F_(1,17)_ = 7.518; *p* = 0.014).

However, whereas GIRK2 levels were decreased in naïve APP_Sw,Ind_ J9 mice compared to naïve WT mice, these changes were not detected after memory training (Figure 4B). These results suggest that spatial memory training was able to restore the expression of this functional subunit in the hippocampus of APP_Sw,Ind_ J9 mice.

## 3. Discussion

It has been previously demonstrated that genetic expression of GIRK in the hippocampus of an amyloidosis mouse model is modulated by the biologically active fragment of amyloid-β (Aβ_25–35_) [20], with deleterious effects on learning and memory processes [30]. The present study aimed to elucidate whether this channel is modulated in a transgenic murine model of AD. Furthermore, we studied the possible effect of age and training in a memory task on this regulation.

Young APP_Sw,Ind_ J9 mice showed a down-regulation of the expression of GIRK2 compared to WT animals. This subunit has been related to several functions, such as learning and memory, reward and motor coordination, and some pathologies like Down syndrome and epilepsy [12,13,31]. Indeed, Down syndrome patients show cerebral accumulation of Aβ and dementia symptoms during aging [32,33,34]. Animals lacking GIRK2 show a reduction of LTP and an increase of long-term depression (LTD) in the hippocampus [13], similarly to what it is observed in AD amyloidopathy [21,22,23]. Since this subunit is believed to be essential for the proper inhibitory function of GIRK channels, its reduction could be contributing to the early hyperexcitability found in this pathology [35]. In fact, GIRK2^−/−^ mice showed elevated motor activity in an open field task and elevated lever press behavior in an operant task [16], which show a hyperactivity in these mice that could be induced by the underlying neural hyperexcitability. In agreement with our results, other authors have shown a reduction in GIRK2 expression in the hippocampus of P301S mice, a murine model of tau pathology, yet no differences were found in APP/PS1 mice [24]. This inconsistency could be due to pathological and/or age differences between the models, since in the present study GIRK2 down-expression was observed only at early ages (6 months) but not in older animals compared to WT, as the ones used by Alfaro-Ruiz et al. [24].

On the other hand, no differences in the expression of any other GIRK subunit were found due to hippocampal accumulation of Aβ (i.e., genotype). Indeed, it has already been reported that amyloidosis did not affect the expression of GIRK1 [23,36]. Available data regarding GIRK3 is limited, however, previous work from our group showed a decrease in gene expression of this subunit induced by soluble Aβ_25–35_ [20], while present results revealed no genotype effect. Other than the possible differences in gene and protein expression, this discrepancy could be due to differences in the experimental design, since the former was an ex vivo Aβ administration in hippocampal slices from 1-month-old rats [20] and the latter is an in vivo Aβ physiological increase in older (6- and 12–18-month-old) mice.

Moreover, our data show an age-dependent decrease in hippocampal GIRK2 expression along with an overexpression of GIRK3, regardless Aβ hippocampal accumulation due to genotype. This age-dependent change in GIRK channel conformation is in line with previous studies, which showed that GIRK2 progressively decreased with age, whereas GIRK1 and GIRK3 gradually increased during postnatal development to reach adult levels [37]. Normal ageing is related to deficits in neurotransmission and synaptic plasticity in several brain areas that translates in the impoverishment of hippocampal-dependent memory processes [38,39,40,41,42,43]. Memory deficits in early AD have been attributed to selective neural impairments at synaptic and network levels induced by soluble Aβ forms as well as neurodegeneration in the entorhinal cortex, CA1 and the subiculum [44,45,46,47]. Although CA1 seems to be the most vulnerable area of the hippocampus, there is almost no neuron loss in this region in normal ageing [48] and no neuronal cell death has been found in APP_Sw,Ind_ J9 mice [49]. Double et al. [50] pointed to GIRK2 as one possible contributor for the selective vulnerability to neural death within different brain regions. For instance, in Parkinson’s disease patients, GIRK2 is mainly expressed in the substantia nigra, where 90% of dopaminergic neurons are lost [50]. Thus, it seems that GIRK2 expression may have a role in the vulnerability of the hippocampus to Aβ.

Interestingly, 6-month-old APP_Sw,Ind_ J9 mice trained in the MWM did not exhibit the decrease in GIRK2 that was observed in naïve transgenic animals. Plenty of studies had shown the benefits of cognitive training to slow down early AD’s symptoms [25,26]. It is relevant that a previous report indicated that, compared with trained control mice, APP_Sw,Ind_ J9 mice showed 932 genes (88% downregulated and 12% upregulated) differentially expressed in the hippocampus. This gene profile revealed a gene cluster of 164 transcripts related to learning/memory, neurotransmission, synaptic plasticity, glutamatergic and GABAergic neurotransmission, oxidative phosphorylation and AD [6,7]. Furthermore, the overexpression of GIRK2 in dorsal CA1 pyramidal neurons was able to restore contextual fear learning in a GIRK2^−/−^ mouse line [51]. In this line, our results indicate that training in a spatial learning task seems to be a good strategy to compensate the GIRK channel expression deficits caused by early Aβ accumulation, probably by counteracting the characteristic hippocampal hyperexcitability present in this pathology [21,22,23]. It remains to be explored whether longer training paradigms could be advantageous to this testing.

GIRK channels, as one of the main determinants of neuronal excitability, support hippocampal-dependent cognitive functions [11], so the decrease in hippocampal GIRK2 protein expression found in J9 mice could underlie synaptic processes impairments leading to early memory deficits in AD mouse models. Moreover, training in a cognitive hippocampal-dependent task reverses this GIRK channel modulation, most likely enhancing its inhibitory activity, and therefore lessening/ameliorating the Aβ mediated excitability impairments present in early AD stages [5,52,53,54,55,56,57,58], as it has been previously observed in amyloidosis models [22,23].

## 4. Materials and Methods 

### 4.1. APP_Sw,Ind_ J9 Transgenic Mice

Male APP_Sw,Ind_ transgenic mice (line J9; C57BL/6 background; *Mus musculus*), that express the human APP695 harboring the FAD-linked Swedish (K670N/M671L) and Indiana (V717F) mutations under the platelet-derived growth factor subunit B (PDGFβ) promoter, were obtained by crossing heterozygous APP_Sw,Ind_ J9 to non-transgenic (WT) mice [3]. Age-matched male WT littermates were used as control (C57BL/6 background). Both WT and APP_Sw;Ind_ J9 mice were genotyped individually. All experimental procedures were conducted according to the approved protocols from the Animal and Human Ethical Committee of the Universitat Autònoma de Barcelona (CEEAH 2895) and Generalitat de Catalunya (10571) following the experimental European Union guidelines and regulations (2010/63/EU).

To assess the effect of age on GIRK expression, littermate WT and APP_Sw;Ind_ J9 mice at 6 months (*n* = 5–8) and 12–18 months (*n* = 3–5) of age were used. Furthermore, to evaluate the effect of learning, 6-month-old APP_Sw;Ind_ J9 and WT naïve mice (*n* = 5–8) were compared to age-matched mice trained in the Morris Water Maze (MWM) (*n* = 4), as previously described [7].

Briefly, as shown in Figure 1, handled mice were trained in a circular pool (90 cm diameter; 6.5 cm hidden platform) for five consecutive days (4 trials daily; 60 s per trial). Memory retention was tested 2.5 h after the last training session in a probe test (without a platform), and mice were sacrificed 30 min afterwards. Naïve groups were placed in the pool (without platform) to freely swim for an equal amount of time and mice were killed 30 min after a simulated probe trial to equalize possible stress levels. Those times were chosen in order to get a measure of memory retention while achieving a maximum peak of gene expression, which occurs about 0.5–2 h after spatial training [7,59].

Animals at the proper age (6- or 12–18-month-old) or 30 min after MWM training were sacrificed by decapitation, hippocampi were dissected, and samples were frozen at −80 °C until further use.

### 4.2. Immunohistochemical Staining

To assess age-dependent amyloid pathology, a protocol shown to label specifically Aβ in APP transgenic mice was used, as described previously [60]. Sagittal brain paraffin sections (5 µm) were deparaffinized in xylene, rehydrated, and incubated with 3% hydrogen peroxide. Sections were then incubated in 60% formic acid for 6 min to allow antigen retrieval, washed in 0.1 M Tris-HCl, and incubated with anti-Aβ (6E10; 1:1000; Signet, Dedham, MA, US) before immunoperoxidase staining and analysis with a Nikon Eclipse 90i microscope.

### 4.3. Western Blot

Whole hippocampal tissue samples were homogenized in ice-cold lysis RIPA-DOC buffer (Sigma-Aldrich, St. Louis, MO, US). Protein concentration was measured using the Pierce™ BCA Protein Assay Kit (Thermo Fisher Scientific, Waltham, MA, US) according to the manufacturer’s instructions. Equal amounts of protein (10 μg) were loaded on an SDS-PAGE gel (10%) and subjected to electrophoresis. Proteins were transferred to PVDF membranes (Bio-Rad, Hercules, CA, US) using a trans-blot turbo apparatus (Bio-Rad, Hercules, CA, US). Membranes were blocked with 5% dried skimmed milk powder in Tween-TBS for 1 h. Primary antibodies (Table 1) were applied at the appropriate dilution overnight at 4 °C. After washing, appropriate secondary antibodies (Table 1) were added for 45 min at a dilution of 1/3000.

Blots were detected after incubation in enhanced chemiluminescence reagent (ECL Prime; Bio-Rad, Hercules, CA, US), using the G:BOX Chemi XX6 system (Syngene, Bangalore, India). In order to check the equal loading of samples, blots were re-incubated with β-actin antibody as a housekeeping gene (Affinity Bioreagents, Golden, CO, US) and data is expressed as the ratio of target protein and β-actin.

### 4.4. Statistical Analysis

Two way-ANOVA was used to assess differences between genotype and age/training. When comparing only two groups, Student’s t test was used. Data is expressed as the mean ± S.E.M., and all analyses were performed using the IBM SPSS Statistics 24 software (SPSS Inc., Chicago, IL, US). A value of *p* < 0.05 was considered statistically significant. Final figures were prepared using CorelDraw v.18 Graphics Suite Software (RRID:SCR_014235).

## Figures and Tables

**Table 1:** Characteristics of primary and secondary antibodies used to measure protein expression by Western Blot.

Protein	Reference	Supplier	Host	Dilution
GIRK1	APC-005	Alomone, Jerusalem, Israel	Rabbit	1/500
GIRK2	APC-006	Alomone, Jerusalem, Israel	Rabbit	1/500
GIRK3	APC-038	Alomone, Jerusalem, Israel	Rabbit	1/500
GIRK4	APC-027	Alomone, Jerusalem, Israel	Rabbit	1/500
RGS7	SC-8139	Santa Cruz Biotech, Dallas, TX, US	Goat	1/500
β-actin	AC-15	Sigma Aldrich, St. Louis, MO, US	Mouse	1:100000
Rabbit IgG-HRP	170-6515	Bio-Rad, Hercules, CA, US	Goat	1/3000
Mouse IgG-HRP	170-6516	Bio-Rad, Hercules, CA, US	Goat	1/3000
Goat IgG-HRP	SC-2352	Merck—Millipore, Burlington, MA, US	Monkey	1/3000

## Abbreviations

Aβ	Amyloid-β
AD	Alzheimer’s disease
APP	Amyloid precursor protein
GIRK	G-protein-gated potassium channels
LTD	Long term depression
LTP	Long term potentiation
MWM	Morris water maze
RSG7	G-protein signaling 7
WT	Wild type

## References

[1] Gauthier S., Rosa-Neto P., Morais J.A., Webster C. (2021). World Alzheimer Report 2021: Journey through the Diagnosis of Dementia.

[2] Jeremic D., Jiménez-Díaz L., Navarro-López J.D. (2021). Past, present and future of therapeutic strategies against amyloid-β peptides in Alzheimer’s disease: A systematic review. Ageing Res. Rev..

[3] Mucke L., Masliah E., Yu G.Q., Mallory M., Rockenstein E.M., Tatsuno G., Hu K., Kholodenko D., Johnson-Wood K., McConlogue L. (2000). High-level neuronal expression of abeta 1-42 in wild-type human amyloid protein precursor transgenic mice: Synaptotoxicity without plaque formation. J. Neurosci..

[4] Chin J., Palop J.J., Puoliväli J., Massaro C., Bien-Ly N., Gerstein H., Scearce-Levie K., Masliah E., Mucke L. (2005). Fyn kinase induces synaptic and cognitive impairments in a transgenic mouse model of Alzheimer’s disease. J. Neurosci..

[5] Palop J.J., Jones B., Kekonius L., Chin J., Yu G.Q., Raber J., Masliah E., Mucke L. (2003). Neuronal depletion of calcium-dependent proteins in the dentate gyrus is tightly linked to Alzheimer’s disease-related cognitive deficits. Proc. Natl. Acad. Sci. USA.

[6] Saura C.A., Parra-Damas A., Enriquez-Barreto L. (2015). Gene expression parallels synaptic excitability and plasticity changes in Alzheimer’s disease. Front. Cell. Neurosci..

[7] Parra-Damas A., Valero J., Chen M., España J., Martín E., Ferrer I., Rodríguez-Alvarez J., Saura C.A. (2014). Crtc1 activates a transcriptional program deregulated at early Alzheimer’s disease-related stages. J. Neurosci..

[8] Mucke L., Selkoe D.J. (2012). Neurotoxicity of amyloid β-protein: Synaptic and network dysfunction. Cold Spring Harb. Perspect. Med..

[9] Hector A., Brouillette J. (2020). Hyperactivity Induced by Soluble Amyloid-β Oligomers in the Early Stages of Alzheimer’s Disease. Front. Mol. Neurosci..

[10] Dascal N. (1997). Signalling via the G protein-activated K^+^ channels. Cell. Signal..

[11] Djebari S., Iborra-Lázaro G., Temprano-Carazo S., Sánchez-Rodríguez I., Nava-Mesa M.O., Múnera A., Gruart A., Delgado-García J.M., Jiménez-Díaz L., Navarro-López J.D. (2021). G-Protein-Gated Inwardly Rectifying Potassium (Kir3/GIRK) Channels Govern Synaptic Plasticity That Supports Hippocampal-Dependent Cognitive Functions in Male Mice. J. Neurosci..

[12] Jeremic D., Sanchez-Rodriguez I., Jimenez-Diaz L., Navarro-Lopez J.D. (2021). Therapeutic potential of targeting G protein-gated inwardly rectifying potassium (GIRK) channels in the central nervous system. Pharmacol. Ther..

[13] Lüscher C., Slesinger P.A. (2010). Emerging roles for G protein-gated inwardly rectifying potassium (GIRK) channels in health and disease. Nat. Rev. Neurosci..

[14] Slesinger P.A., Wickman K., Slesinger P.A., Wickman K. (2015). Structure to Function of G Protein-Gated Inwardly Rectifying (GIRK) Channels..

[15] Karschin C., Dissmann E., Stühmer W., Karschin A. (1996). IRK(1-3) and GIRK(1-4) inwardly rectifying K^+^ channel mRNAs are differentially expressed in the adult rat brain. J. Neurosci..

[16] Pravetoni M., Wickman K. (2008). Behavioral characterization of mice lacking GIRK/Kir3 channel subunits. Genes Brain Behav..

[17] Ostrovskaya O.I., Orlandi C., Fajardo-Serrano A., Young S.M., Lujan R., Martemyanov K.A. (2018). Inhibitory Signaling to Ion Channels in Hippocampal Neurons Is Differentially Regulated by Alternative Macromolecular Complexes of RGS7. J. Neurosci..

[18] Ostrovskaya O., Xie K., Masuho I., Fajardo-Serrano A., Lujan R., Wickman K., Martemyanov K.A. (2014). RGS7/Gβ5/R7BP complex regulates synaptic plasticity and memory by modulating hippocampal GABABR-GIRK signaling. Elife.

[19] Nava-Mesa M.O., Jiménez-Díaz L., Yajeya J., Navarro-Lopez J.D. (2013). Amyloid-β induces synaptic dysfunction through G protein-gated inwardly rectifying potassium channels in the fimbria-CA3 hippocampal synapse. Front. Cell. Neurosci..

[20] Mayordomo-Cava J., Yajeya J., Navarro-López J.D., Jiménez-Díaz L. (2015). Amyloid-β(25-35) Modulates the Expression of GirK and KCNQ Channel Genes in the Hippocampus. PLoS ONE.

[21] Sánchez-Rodríguez I., Gruart A., Delgado-García J.M., Jiménez-Díaz L., Navarro-López J.D. (2019). Role of GirK Channels in Long-Term Potentiation of Synaptic Inhibition in an In Vivo Mouse Model of Early Amyloid-β Pathology. Int. J. Mol. Sci..

[22] Sánchez-Rodríguez I., Temprano-Carazo S., Nájera A., Djebari S., Yajeya J., Gruart A., Delgado-García J.M., Jiménez-Díaz L., Navarro-López J.D. (2017). Activation of G-protein-gated inwardly rectifying potassium (Kir3/GirK) channels rescues hippocampal functions in a mouse model of early amyloid-β pathology. Sci. Rep..

[23] Sánchez-Rodríguez I., Djebari S., Temprano-Carazo S., Vega-Avelaira D., Jiménez-Herrera R., Iborra-Lázaro G., Yajeya J., Jiménez-Díaz L., Navarro-López J.D. (2019). Hippocampal long-term synaptic depression and memory deficits induced in early amyloidopathy are prevented by enhancing G-protein-gated inwardly rectifying potassium channel activity. J. Neurochem..

[24] Alfaro-Ruiz R., Martín-Belmonte A., Aguado C., Hernández F., Moreno-Martínez A.E., Ávila J., Luján R. (2021). The Expression and Localisation of G-Protein-Coupled Inwardly Rectifying Potassium (GIRK) Channels Is Differentially Altered in the Hippocampus of Two Mouse Models of Alzheimer’s Disease. Int. J. Mol. Sci..

[25] Rai S.P., Krohn M., Pahnke J. (2020). Early Cognitive Training Rescues Remote Spatial Memory but Reduces Cognitive Flexibility in Alzheimer’s Disease Mice. J. Alzheimer’s Dis..

[26] Martinez-Coria H., Yeung S.T., Ager R.R., Rodriguez-Ortiz C.J., Baglietto-Vargas D., LaFerla F.M. (2015). Repeated cognitive stimulation alleviates memory impairments in an Alzheimer’s disease mouse model. Brain Res. Bull..

[27] Oveisgharan S., Wilson R.S., Yu L., Schneider J.A., Bennett D.A. (2020). Association of Early-Life Cognitive Enrichment With Alzheimer Disease Pathological Changes and Cognitive Decline. JAMA Neurol..

[28] Hall C.B., Lipton R.B., Sliwinski M., Katz M.J., Derby C.A., Verghese J. (2009). Cognitive activities delay onset of memory decline in persons who develop dementia. Neurology.

[29] Zhao Y., Bao J., Liu W., Gong X., Liang Z., Li W., Wu M., Xiao Y., Sun B., Wang X. (2022). Spatial Training Attenuates Long-Term Alzheimer’s Disease-Related Pathogenic Processes in APP/PS1 Mice. J. Alzheimer’s Dis..

[30] Mayordomo-Cava J., Iborra-Lázaro G., Djebari S., Temprano-Carazo S., Sánchez-Rodríguez I., Jeremic D., Gruart A., Delgado-García J.M., Jiménez-Díaz L., Navarro-López J.D. (2020). Impairments of Synaptic Plasticity Induction Threshold and Network Oscillatory Activity in the Hippocampus Underlie Memory Deficits in a Non-Transgenic Mouse Model of Amyloidosis. Biology.

[31] Luján R., Marron Fernandez de Velasco E., Aguado C., Wickman K. (2014). New insights into the therapeutic potential of Girk channels. Trends Neurosci..

[32] Cooper A., Grigoryan G., Guy-David L., Tsoory M.M., Chen A., Reuveny E. (2012). Trisomy of the G protein-coupled K^+^ channel gene, Kcnj6, affects reward mechanisms, cognitive functions, and synaptic plasticity in mice. Proc. Natl. Acad. Sci. USA.

[33] Moncaster J.A., Pineda R., Moir R.D., Lu S., Burton M.A., Ghosh J.G., Ericsson M., Soscia S.J., Mocofanescu A., Folkerth R.D. (2010). Alzheimer’s disease amyloid-beta links lens and brain pathology in Down syndrome. PLoS ONE.

[34] Lott I.T., Head E. (2005). Alzheimer disease and Down syndrome: Factors in pathogenesis. Neurobiol. Aging.

[35] Sanchez P.E., Zhu L., Verret L., Vossel K.A., Orr A.G., Cirrito J.R., Devidze N., Ho K., Yu G.Q., Palop J.J. (2012). Levetiracetam suppresses neuronal network dysfunction and reverses synaptic and cognitive deficits in an Alzheimer’s disease model. Proc. Natl. Acad. Sci. USA.

[36] Akyuz E., Villa C., Beker M., Elibol B. (2020). Unraveling the Role of Inwardly Rectifying Potassium Channels in the Hippocampus of an Aβ((1-42))-Infused Rat Model of Alzheimer’s Disease. Biomedicines.

[37] Fernández-Alacid L., Watanabe M., Molnár E., Wickman K., Luján R. (2011). Developmental regulation of G protein-gated inwardly-rectifying K^+^ (GIRK/Kir3) channel subunits in the brain. Eur J. Neurosci..

[38] Disterhoft J.F., Wu W.W., Ohno M. (2004). Biophysical alterations of hippocampal pyramidal neurons in learning, ageing and Alzheimer’s disease. Ageing Res. Rev..

[39] Barnes C.A. (2003). Long-term potentiation and the ageing brain. Philos. Trans. R. Soc. B Biol. Sci..

[40] Foster T.C., Norris C.M. (1997). Age-associated changes in Ca(2+)-dependent processes: Relation to hippocampal synaptic plasticity. Hippocampus.

[41] Cohen S.J., Stackman R.W. (2015). Assessing rodent hippocampal involvement in the novel object recognition task. A review. Behav. Brain Res..

[42] Clarke J.R., Cammarota M., Gruart A., Izquierdo I., Delgado-García J.M. (2010). Plastic modifications induced by object recognition memory processing. Proc. Natl. Acad. Sci. USA.

[43] Warburton E.C., Brown M.W. (2010). Findings from animals concerning when interactions between perirhinal cortex, hippocampus and medial prefrontal cortex are necessary for recognition memory. Neuropsychologia.

[44] Giannakopoulos P., Gold G., von Gunten A., Hof P.R., Bouras C. (2009). Pathological substrates of cognitive decline in Alzheimer’s disease. Front. Neurol. Neurosci..

[45] Morrison J.H., Hof P.R. (2002). Selective vulnerability of corticocortical and hippocampal circuits in aging and Alzheimer’s disease. Prog. Brain Res..

[46] Li S., Hong S., Shepardson N.E., Walsh D.M., Shankar G.M., Selkoe D. (2009). Soluble oligomers of amyloid Beta protein facilitate hippocampal long-term depression by disrupting neuronal glutamate uptake. Neuron.

[47] Forloni G., Lucca E., Angeretti N., Della Torre P., Salmona M. (1997). Amidation of beta-amyloid peptide strongly reduced the amyloidogenic activity without alteration of the neurotoxicity. J. Neurochem..

[48] West M.J., Coleman P.D., Flood D.G., Troncoso J.C. (1994). Differences in the pattern of hippocampal neuronal loss in normal ageing and Alzheimer’s disease. Lancet.

[49] Balducci C., Forloni G. (2011). APP transgenic mice: Their use and limitations. Neuromolecular Med..

[50] Double K.L., Reyes S., Werry E.L., Halliday G.M. (2010). Selective cell death in neurodegeneration: Why are some neurons spared in vulnerable regions?. Prog. Neurobiol..

[51] Marron Fernandez de Velasco E., Zhang L., Vo B.N., Tipps M., Farris S., Xia Z., Anderson A., Carlblom N., Weaver C.D., Dudek S.M. (2017). GIRK2 splice variants and neuronal G protein-gated K(+) channels: Implications for channel function and behavior. Sci. Rep..

[52] Hall A.M., Throesch B.T., Buckingham S.C., Markwardt S.J., Peng Y., Wang Q., Hoffman D.A., Roberson E.D. (2015). Tau-dependent Kv4.2 depletion and dendritic hyperexcitability in a mouse model of Alzheimer’s disease. J. Neurosci..

[53] Minkeviciene R., Rheims S., Dobszay M.B., Zilberter M., Hartikainen J., Fülöp L., Penke B., Zilberter Y., Harkany T., Pitkänen A. (2009). Amyloid beta-induced neuronal hyperexcitability triggers progressive epilepsy. J. Neurosci..

[54] Toniolo S., Sen A., Husain M. (2020). Modulation of Brain Hyperexcitability: Potential New Therapeutic Approaches in Alzheimer’s Disease. Int. J. Mol. Sci..

[55] Busche M.A., Konnerth A. (2015). Neuronal hyperactivity--A key defect in Alzheimer’s disease?. Bioessays.

[56] Maestú F., de Haan W., Busche M.A., DeFelipe J. (2021). Neuronal excitation/inhibition imbalance: Core element of a translational perspective on Alzheimer pathophysiology. Ageing Res. Rev..

[57] Zott B., Busche M.A., Sperling R.A., Konnerth A. (2018). What Happens with the Circuit in Alzheimer’s Disease in Mice and Humans?. Annu Rev. Neurosci..

[58] Verret L., Mann E.O., Hang G.B., Barth A.M., Cobos I., Ho K., Devidze N., Masliah E., Kreitzer A.C., Mody I. (2012). Inhibitory interneuron deficit links altered network activity and cognitive dysfunction in Alzheimer model. Cell.

[59] Guzowski J.F., Setlow B., Wagner E.K., McGaugh J.L. (2001). Experience-dependent gene expression in the rat hippocampus after spatial learning: A comparison of the immediate-early genes Arc, c-fos, and zif268. J. Neurosci..

[60] España J., Giménez-Llort L., Valero J., Miñano A., Rábano A., Rodriguez-Alvarez J., LaFerla F.M., Saura C.A. (2010). Intraneuronal beta-amyloid accumulation in the amygdala enhances fear and anxiety in Alzheimer’s disease transgenic mice. Biol. Psychiatry.

